# Integrating Full-Length and Second-Generation Transcriptomics to Reveal Differentially Expressed Genes Associated with the Development of *Corydalis yanhusuo* Tuber

**DOI:** 10.3390/life13112207

**Published:** 2023-11-14

**Authors:** Xueyan Zhao, Li Wang, Yafu Zhou, Qing Wang, Fangyuan Wang, Yan Li

**Affiliations:** Shaanxi Engineering Research Centre for Conservation and Utilization of Botanical Resources, Xi’an Botanical Garden of Shaanxi Province (Institute of Botany of Shaanxi Province), Xi’an 710061, Chinawangli_8587@163.com (L.W.); zyf820207@126.com (Y.Z.); icely415@163.com (Q.W.); wangfy0125@163.com (F.W.)

**Keywords:** *Corydalis yanhusuo*, transcriptomics, differentially expressed genes, tuber development

## Abstract

*Corydalis yanhusuo* is a medicinal herb in China that has been widely used to treat various kinds of pain. The tuber is the main organ of *C. yanhusuo* used for medicinal purposes, but changes in related genes during the development of the tuber have rarely been reported. To identify the differentially expressed genes during tuber development, *C. yanhusuo* full-length transcriptomic sequencing was performed using single-molecule real-time technology, and tubers at three development stages were selected for comparative transcriptome analysis. A total of 90,496 full-length non-chimeric transcripts were obtained, and 19,341 transcripts were annotated in at least one public database. A total of 9221 differentially expressed genes were identified during the swelling process of *C. yanhusuo* tuber. A Kyoto encyclopedia of genes and genomes (KEGG) pathway enrichment analysis revealed that differentially expressed genes associated with a “starch and sucrose metabolism pathway”, “phenylpropanoid biosynthesis pathway”, “isoquinoline alkaloid biosynthesis pathway”, “zeatin biosynthesis pathway”, and “brassinosteroid biosynthesis pathway” were predominantly enriched. In addition, the genes involved in cell wall metabolism were potentially associated with tuber swelling. These processes regulated and were involved in *C. yanhusuo* tuber development. The results provide a foundation for further research on tuber formation in medicinal plants.

## 1. Introduction

*Corydalis yanhusuo* W.T. Wang is a medicinal herb in the *Corydalis* genus of the Papaveraceae family. *C. yanhusuo* contains several kinds of chemical components, including alkaloids, sugars, amino acid derivatives, triterpenes, anthraquinones, phenolic acids, steroids, and organic acids [1,2], and alkaloids are known to be its major active compound. The dried tuber of *C. yanhusuo* is a commonly used and well-known traditional Chinese medicine [3] and can be used for pain relief [4]. Pain can cause discomfort, which includes physiological and psychological changes, and may ultimately lead to disease, disability, or death [5]. *C. yanhusuo* effectively attenuates acute, inflammatory, and neuropathic pain without causing tolerance [6]. In addition, it was reported that *C. yanhusuo* has shown anti-tumor [7], anti-anxiety [1], anti-myocardial ischemia [8], neuraminidase inhibitory [9], and anti-platelet aggregation activities [10], and it was often used to treat miscellaneous medical disorders such as insomnia, cardiovascular diseases, hypertension, gastric ulcers, cancer, and inflammation [11].

The enlarged root, tuber, rhizome, and tuberous root of medicinal plants are important medicinal organs that accumulate numerous active ingredients. The formation of storage organs is induced by environmental and endogenous factors, such as photoperiod, high sucrose, and hormone changes [12,13]. These organs’ thickening is mainly regulated by the vascular system. The tuber of *C. yanhusuo* is derived from the expansion of the underground stem node. Firstly, an irregular vascular cambium originates from the other side of the stem node, expands continuously, and finally, the annular vascular cambium is formed with the development of the tuber. Afterwards, the cambium cells divide continuously, which produces a mass of secondary phloem cells outward and secondary xylem cells inward that lead to a rapid expansion of the tuber [14]. Currently, the developmental process of the *C. yanhusuo* tuber at the molecular level is less well characterized.

Recently, single-molecule real-time (SMRT) technology carried out in PacBio RS has been used to obtain full-length transcripts that do not need to be assembled. The accuracy of SMRT sequencing can be addressed by second-generation sequencing of short-read data [15]. SMRT sequencing can offer highly complete transcript data for further analysis of the exon–intron structure and alternative splicing events [16]. Therefore, third-generation sequencing has been used to analyze full-length transcripts in medicinal plants, for instance, *Salvia miltiorrhiza* [17], *Carthamus tinctorius* [18], and *Coptis deltoidea* [19].

The life cycle of *C. yanhusuo* is short, and the development of the tuber greatly affects its productivity. In addition, the development of the tuber is related to the accumulation of isoquinoline alkaloids [20], thus directly affecting its quality. To date, research on *C. yanhusuo* has mainly focused on the pharmacological effects and the separation of compounds. Transcriptome studies of *C. yanhusuo* have been carried out, which were performed to identify the genes related to secondary metabolite biosynthesis, and 187 candidate genes and 101 full-length transcripts were identified, respectively, which involved benzylisoquinoline alkaloids [21,22].

The tuber of *C. yanhusuo* is used for treating pain, and the yield is the key factor in its economic value. Nevertheless, changes during tuber development have not been reported. *C. yanhusuo* tubers have three pronounced periods of change: the formation of the tuber (initial expanding stage), the swelling of the tuber (rapid swelling stage), and tuber maturation (maturation stage). In order to understand differentially expressed genes and the metabolic pathways involved in *C. yanhusuo* tuber development, full-length transcriptomic sequencing was performed by the SMRT sequencing technique on the PacBio Sequel platform, and tubers at the three different development stages were selected to be analyzed by the second-generation sequencing technique. The results reported in this paper will provide full-length transcriptome information on *C. yanhusuo* and an improved understanding of the mechanisms underlying *C. yanhusuo* tuber development.

## 2. Materials and Methods

### 2.1. Plant Materials

*C. yanhusuo* plants were planted in an experimental field of Xi’an Botanical Garden, Shaanxi province, China (34.21 N, 108.95 E). Leaves, rhizomes, and tubers at three different development stages (C01, initial expanding stage; C02, rapid swelling stage; C03, maturation stage) were collected, quickly frozen in liquid nitrogen, and stored at −80 °C until RNA extraction.

### 2.2. RNA Extraction, Iso-Seq Library Construction, and Single-Molecular Real-Time Sequencing

Total RNA from leaves, rhizomes, and tubers at three different development stages of *C. yanhusuo* was extracted using the TRIzol reagent (Invitrogen, Carlsbad, CA, USA) according to the manufacturer’s protocol. The same amount of RNA from leaves, rhizomes, and tubers at three different development stages was mixed for full-length sequencing analysis. The purity, concentration, and integrity of the total RNA were determined by 1% agarose gel electrophoresis, NanoDrop spectrophotometer (NanoDrop Technologies, Wilmington, DE, USA), and Agilent 2100 Bioanalyzer.

The qualified RNA samples were reversely transcribed into cDNA using a SMARTer™ PCR cDNA Synthesis Kit (Takara Bio, Mountain View, CA, USA). Then, PCR was used to amplify the full-length cDNA. The end of the full-length cDNA was repaired and connected to the SMRT dumbbell-type connector. The library was obtained after exonuclease treatment. After construction of the Iso-seq library, SMRT sequencing was performed on a Pacific Bioscience platform.

### 2.3. Analysis of the Full-Length Transcriptome

The analysis of the full-length transcriptome consisted of three stages: full-length sequence recognition, isoform-level clustering to obtain a consistent sequence, and a consistent sequence of polishing [23]. First, raw reads were processed into circular consensus sequencing (CCS) read according to the adapter, and then the CCS were divided into full-length and non-full-length sequences according to whether they contained the 3ʹ-primer, 5ʹ-primer, and polyA (optional). Then, by clustering the similar full-length sequences from the same transcript into a cluster, a consistent sequence was obtained from each cluster. Lastly, the high-quality isoforms were obtained by polishing consensus isoforms and used for subsequent analysis.

### 2.4. Gene Functional Annotation

The full-length non-chimeric transcripts were annotated using BLAST (version 2.2.26) [24] in the public databases, which contained Nr (NCBI non-redundant protein sequences) [25], Pfam (Protein family) [26], KOG/COG (Clusters of Orthologous Groups of proteins) [27,28], Swiss-Prot (a manually annotated and reviewed protein sequence database) [29], KEGG [30], and GO (Gene Ontology) [31].

### 2.5. Illumina cDNA Library Construction and Second-Generation Sequencing

Tubers at three different development stages of *C. yanhusuo* were used for second-generation sequencing. Each sample had three biological replicates. After the total RNA was extracted, the mRNA was enriched by Oligo (dT) beads. The enriched mRNA was fragmented into short fragments using a fragmentation buffer and then reverse transcribed into cDNA with random primers. Second-strand cDNA was synthesized by DNA polymerase I, RNase H, dNTPs, and the buffer. The cDNA fragments were purified using an AMPure XP kit following end-repair and the addition of polyA. Then, Illumina adapters were ligated to sequence. The cDNA fragments were purified and enriched by PCR to construct the final cDNA library. Finally, the cDNA library was sequenced on the Illumina sequencing platform.

The obtained raw data of fastq format were first processed through in-house perl scripts. Clean reads were obtained by removing reads containing adapter, ploy-N, and low-quality reads. Q30 and GC contents of the clean data were calculated. The clean data yielded by Illumina were further used for correction of the PacBio sequencing data.

### 2.6. Analysis of Differentially Expressed Genes

Gene expression levels were estimated by fragments per kilobase of transcript per million fragments mapped. Differential expression analysis of two samples was performed using the edgeR package (http://www.r-project.org/, accessed on 10 July 2020). The resulting *p*-values were adjusted using Benjamini and Hochberg’s approach for controlling the false discovery rate. Genes with a false discovery rate (*FDR*) < 0.01 and fold change ≥ 2 were assigned as differentially expressed genes (DEGs).

### 2.7. GO and KEGG Pathway Enrichment Analysis of Differentially Expressed Genes

GO enrichment analysis of DEGs was implemented by the GOseq R packages which was based Wallenius non-central hyper-geometric distribution, which could adjust for gene length bias in DEGs. GO terms with corrected *p*-values < 0.05 were considered significantly enriched. Afterwards, KEGG pathway enrichment analysis was performed by KOBAS 3.0 software to test the statistical enrichment of DEGs in KEGG pathways with an *FDR* < 0.05 [32].

### 2.8. Quantitative Real-Time PCR Analysis

RNase-free DNase I (Takara Biotechnology, Dalian, China) was used to pretreat RNA samples before their use in reverse transcription to minimize DNA contamination. Complementary DNA was synthesized with the TRUEscript 1st Strand cDNA Synthesis Kit. The used primer pairs used are shown in Table S2. The relative expression levels of the target genes were calculated using the 2^−ΔΔCT^ approach with *actin* as the reference gene. Each gene was analyzed in three biological replicates.

## 3. Results

### 3.1. Morphological Changes during C. yanhusuo Tuber Expanding

**Figure 1:**
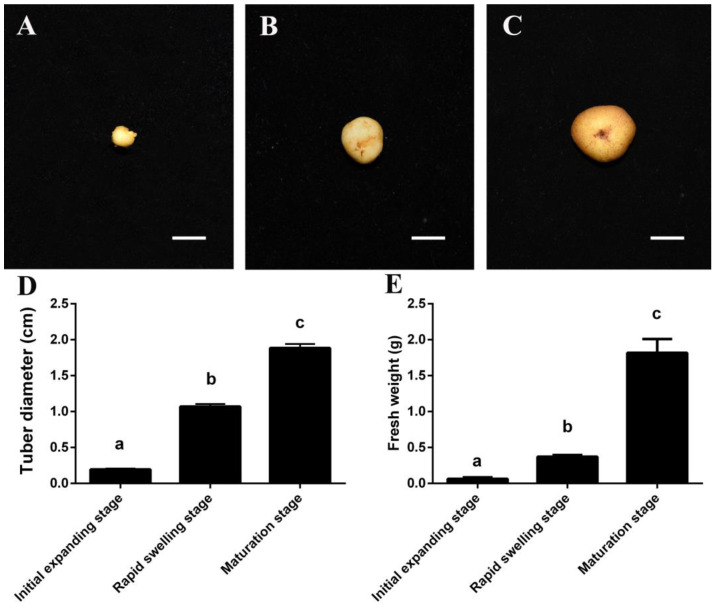
The tuber and determination of growth indices of *Corydalis yanhusuo* at three different development stages. (**A**) Initial expanding stage. (**B**) Rapid swelling stage. (**C**) Maturation stage. (**D**) Tuber diameter. (**E**) Tuber fresh weight. Columns with different letters are significantly different at *p* < 0.05 (*n* = 10).

The tuber expansion of *C. yanhusuo* took place in the middle of March (regarding the initial expanding stage, C01). The stem nodes of the rhizome were just beginning to swell, and the initial cambium was formed (Figure 1A and Figure S1a). While the tuber rapidly swelled in early April (regarding the rapid swelling stage, C02), the annular cambium formed, and the vascular cambium cells divided continuously, which generated a large number of cells and tissues at this stage (Figure 1B and  Figure S1b). The tuber matured at the end of April (regarding the maturation stage, C03) (Figure 1C). The dynamic growth indexes of *C. yanhusuo* tuber were measured. The results showed that the tuber diameter gradually increased, and there were significant differences among the initial expanding stage, rapid swelling stage, and maturation stage (Figure 1D). Similarly, the fresh weight of the tuber increased with the growth period, and there was a significant difference among these three development stages (Figure 1E).

### 3.2. Full-Length Transcriptome Sequencing and Analysis

**Figure 2:**
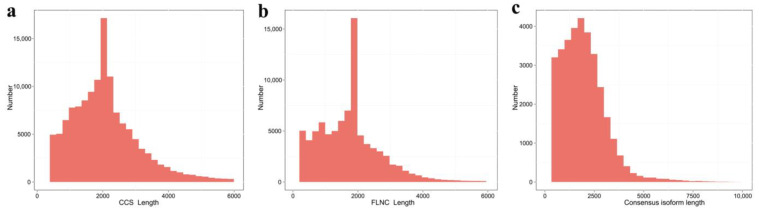
The quality control data for the full-length transcriptome of *Corydalis yanhusuo* obtained by SMRT sequencing. (**a**) The distribution of circular consensus sequences (CCS) length. (**b**) The length distribution of full-length nonchimeric (FLNC) reads. (**c**) The quality of consensus isoform sequences length.

In the present study, the RNA from leaves, rhizomes, and tubers at three different development stages was mixed, and the full-length transcriptome was performed by SMRT sequencing on the PacBio Sequel platform. A total of 136,469 circular consensus sequences (CCS) were obtained with read bases of 288,681,328 bp and a mean read length of 2115 bp. Subsequently, 90,496 full-length non-chimeric (FLNC) reads were detected by identifying the coexistence of 5′-primers, 3′-primers, and poly-A tails, and the percentage of full-length non-chimeric was 66.31%. Similar sequences of the full-length non-chimeric reads were clustered, and 36,347 consensus isoforms were obtained. Of these, 35,513 isoforms (97.71%) were polished high-quality isoforms (Table 1, Figure 2).

### 3.3. Function Annotation of Unigenes

**Figure 3:**
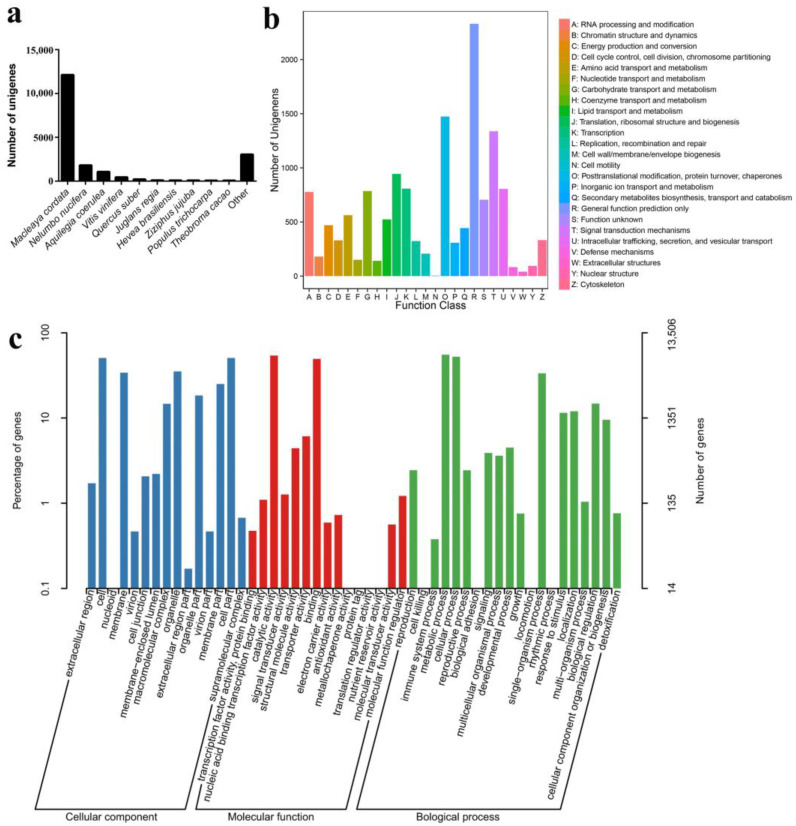
Function annotation of unigenes in *Corydalis yanhusuo*. (**a**) Nr homologous species distribution of unigene annotation; the ten top species are listed. (**b**) Function classification of *C. yanhusuo* unigenes in the KOG database. (**c**) Gene ontology classification of *C. yanhusuo* unigenes.

In order to analyze the functions of the obtained full-length transcripts, we annotated the transcripts in the Nr, Swiss-Prot, KOG, COG, Pfam, GO, and KEGG databases. A total of 19,341 transcripts were annotated in at least one database (Table 2). Based on the Nr database, the species distribution showed that 12,137 (63.11%) of the mapped unigenes were annotated with *Macleaya cordata* homologs, 1819 (9.46%) with *Nelumbo nucifera*, and 1085 (5.64%) with *Aquilegia coerulea*. Lower numbers of unigenes were annotated for *Populus trichocarpa* (86; 0.45%) and *Theobroma cacao* (81; 0.42%) (Figure 3a).

In terms of the KOG annotation, 12,663 unigenes were annotated and assigned to 25 function categories. The “general functional prediction only” group (2331; 18.41%) was the largest group, followed by “post-translational modification, protein turnover, chaperones” (1474; 18.49%) and “signal transduction mechanisms” (1338; 10.57%), whereas only a few unigenes (4; 0.03%) were classified as “cell motility” (Figure 3b).

In the GO dabatase, 13,506 unigenes were assigned to 51 GO categories under the three main categories: biological process, cellular component, and molecular function. Under biological process, 7456 (55.21%) unigenes were assigned to “metabolic process”, followed by “cellular process” (7060; 52.27%). For the cellular component class, the “cell” (6835; 50.61%) and “cell part” (6819; 50.49%) were the dominant terms. For the molecular function category, the major terms were “catalytic activity” (7292; 53.99%) and “binding” (6645; 49.20%) (Figure 3c).

### 3.4. Differential Expression of Genes among C. yanhusuo Tubers at Different Developmental Stages

To investigate the genes related to tuber development, the tubers at the initial expanding stage, rapid swelling stage, and maturation stage were collected and sequenced using second-generation sequencing on the Illumina platform. Each sample had three replicates. 19,850,543–24,651,686 clean reads were obtained, with an average of 21,243,334 clean reads (Table 3). The Q30 percentage of each sample was above 92.72%. The sequencing quality of samples is shown in Figure S2.

**Figure 4:**
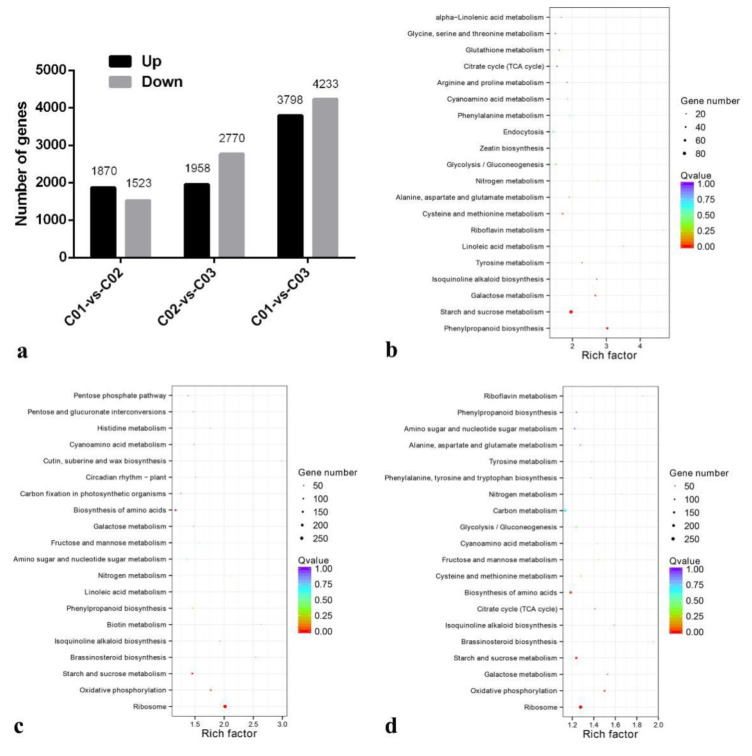
Differentially expressed gene analysis. (**a**) DEGs in different pairwise comparison. (**b**) Pathway enrichment analysis in C01 vs. C02. (**c**) Pathway enrichment analysis in C02 vs. C03. (**d**) Pathway enrichment analysis in C01 vs. C03. C01: initial expanding stage; C02: rapid swelling stage; C03: maturation stage.

In order to obtain reliable gene expression profiles, the unigenes with a fold change ≥ 2 and *FDR* < 0.01 were selected as DEGs. After searching the DEGs in pairwise comparisons of C01 vs. C02, C01 vs. C03, and C02 vs. C03, a total of 9221 DEGs were identified. Compared with C01, 3393 unigenes were regulated in C02, including 1870 up-regulated DEGs and 1523 down-regulated DEGs, and 8031 DEGs were identified (3798 up-regulated and 4233 down-regulated) in C03. Compared with C02, 4728 unigenes were regulated in C03, including 1958 up-regulated DEGs and 2770 down-regulated DEGs (Figure 4a). In addition, 1117 common unigenes were identified in all three pairwise expressions and shown in the Venn diagram (Table S1, Figure S3).

Eight DEGs (1,4-alpha-glucan-branching enzyme, alpha-1,4 glucan phosphorylase, beta-amylase, zeatin O-glucosyltransferase, auxin response factor, peroxidase, galacturonosyltransferase, and pectinesterase) were selected for the quantitative real-time PCR (qRT-PCR) analysis. These differentially expressed genes were involved in starch and sucrose metabolism, hormone biosynthesis and signaling transduction, and cell wall metabolism. The expression profiles of these eight unigenes using qRT-PCR were strongly consistent with those obtained from RNA-Seq (Figure S4). These results indicated that the data from the transcriptome were reliable.

### 3.5. The Function Annotation and Enrichment Analysis of Differential Expression Genes

The DEGs were further analyzed and categorized in databases. As shown in Table 4, a total of 3223, 4453, and 7649 unigenes were, respectively, annotated in sample pairs C01 vs. C02, C01 vs. C03, and C02 vs. C03. In addition, GO enrichment analysis showed that the DEGs were mainly involved in the “metabolic process”, followed by the “cell process” and the “single-organism process” (Figure S5). Metabolic process analysis by the KEGG pathway showed that the DEGs were predominantly enriched in the “starch and sucrose metabolism pathway”, “phenylpropanoid biosynthesis pathway”, “galactose metabolism”, “isoquinoline alkaloid biosynthesis pathway”, “zeatin biosynthesis pathway”, and “brassinosteroid biosynthesis pathway” (Figure 4b–d).

In this study, as the *C. yanhusuo* tubers developed, 119 unigenes involved in starch and sucrose metabolism showed differential expression (Figure S6), and most genes were up-regulated in the rapid swelling stage (C02) and maturation stage (C03). In addition, a total of 80 unigenes related to plant hormone signal transduction were differentially expressed in three developmental stages (Figure S7). The results suggested that these biological pathways were closely related to the development of the *C. yanhusuo* tuber.

## 4. Discussion

### 4.1. Characterization of the C. yanhusuo Full-Length Transcriptome

The tuber of *Corydalis yanhusuo* can be used in traditional Chinese medicine, which has significant analgesic effects [33]. To date, previous transcriptome research on *C. yanhusuo* has focused on identifying candidate genes that might be involved in benzylisoquinoline alkaloid biosynthesis [21,22]. The development of the tuber affects the yield of *Corydalis corydalis*. In addition, Liao et al. (2016) reported that the content of alkaloids also increased with the development of the tuber [21]. In order to obtain more information on tuber development, we performed the full-length transcriptome sequencing based on SMRT technology and comparative transcriptome of tubers at three different developmental stages using second-generation sequencing technology.

In the present research, 90,496 FLNC reads were obtained from three different developmental stages, and 19,341 transcripts were annotated in the public databases. Xu et al. (2021) detected 184,584 FLNC reads from tubers and leaves of *C. yanhusuo* [22]. The obtained full-length transcripts from different tissues can provide more information about *C. yanhusuo*. The obtained full-length transcriptome data may provide more reliable and complete mRNA information in the organism [34,35]. A total of 63.11% of the unigenes were annotated with *M. cordata*. The results showed that most of the genes of *C. yanhusuo* were annotated in *M. cordata,* which also belongs to the Papaveraceae family. This suggests that the sequencing results are credible.

### 4.2. Differential Expression Genes in C. yanhusuo Tuber Swelling

The diameter of the *C. yanhusuo* tuber increased with the development process (Figure 1D). The enlargement of the tuber was associated with cell division of the vascular cambium, whereafter the produced cells continue to divide and differentiate, and this process results in a rapid increase in the tuber diameter. This process is controlled by plant hormones and metabolic pathways [36,37]. As initial and rapid expansion stages are the key stages for tuber development, the consistently up-regulated or down-regulated genes play an important role in tuber development in *C. yanhusuo*.

### 4.3. Differential Expression of Genes Related to Starch and Sucrose Metabolism

“Starch and sucrose metabolism” was the main enrichment KEGG pathway of DEGs in three pairwise comparisons. Starch is considered to be one of the major storage carbohydrates. The swelling of the storage organs was accompanied by the accumulation of starch in potato (*Solanum tuberosum*), lotus (*Nelumbo nucifera*), and cassava (*Manihot esculenta*) [38,39,40]. In the present study, the unigenes involved in starch and sucrose metabolism showed differential expression (Figure S6), and most genes were up-regulated in the rapid swelling stage and maturation stage, including alpha amylase, beta-amylase genes, glycosyl transferase, alpha-1,4 glucan phosphorylase, 1,4-alpha-glucan-branching enzyme, and sucrose synthase genes. This suggests that sucrose metabolism and starch accumulation are required for the swelling of *C. yanhusuo* tubers and play a major role in the rapid swelling and maturation stages. This was consistent with the finding that starch content was highest in the mature stage, and the content of starch granules was related to the content of alkaloids [20]. Liao et al. (2016) found carbohydrate metabolic processes were mainly enriched on the bulbs on day 10 and day 30 of *C. yanhusuo* [21]. Likewise, sucrose synthase was a key enzyme involved in the early development of *Panax notoginseng* taproot thickening [36]. Yang et al. (2011) found that the starch-branching enzyme and glucan phosphorylase for sucrose and starch metabolism were differentially expressed in three developmental phases of roots and indicated that these were the key enzymes required for starch accumulation in the cassava storage root [41]. These indicated that tuber enlargement and maturation were associated with storage metabolism.

### 4.4. Differential Expression of Genes Related to Hormone Biosynthesis and Signaling Transduction

Plant hormones, such as indole-3-acetic acid (IAA), cytokinin (CTK), gibberellin (GA), ethylene (ETH), jasmonate (JA), and brassinosteroid (BR), and the underlying genes have important roles in the initiation and development of plant organs [42,43,44,45]. Liao et al. (2016) found that unigenes involved in plant hormone signal transduction were significantly enriched on the *C. yanhusuo* tuber of day 10 and day 30, suggesting that phytohormones play important roles in regulating the initiation and enlargement of tubers [21].

In addition, KEGG enrichment analysis of DEGs in three pairwise comparisons showed that “brassinosteroid biosynthesis” and “zeatin biosynthesis” were predominantly enriched during tuber development. Brassinosteroids are ubiquitous plant hormones that promote plant growth and developmental processes by regulating cell elongation, division, and differentiation [46,47]. Cytochrome P450 genes, which are involved in brassinosteroid biosynthesis, were significantly up-regulated with tuber development (C02 and C03). The results suggested that brassinosteroids might play an important role in the swelling of *C. yanhusuo* tuber. Zeatin is a kind of typical cytokinin that plays important roles in regulating the proliferation and differentiation of plant cells and is widely distributed in various plant tissues [48]. The DEGs, including zeatin O-glucosyltransferase, which is related to zeatin biosynthesis, were up-regulated in the early stage and down-regulated in maturation stage. Zeatin may promote the formation of *C. yanhusuo* tuber. Similarly, the genes related to zeatin synthesis were highly expressed in tuberous formation stage of *Tetrastigma hemsleyanum* [49]. Furthermore, most DEGs associated with the IAA signaling pathway were up-regulated mainly in the maturation stage, including auxin response factor 5, auxin response factor 9, and auxin-responsive protein IAA17. These results indicate that plant hormones may play different functions at three developmental stages of tuber.

### 4.5. Differential Expression of Genes Related to Cell Wall Metabolism

The genes involved in cell wall modification, synthesis, and degradation metabolism have been analyzed in the present study. Among them, the largest number of genes, such as pectinesterase, sucrose synthase, and 14-3-3 protein, had higher expression level in the initial expanding stage of the tuber, suggesting that the changes in the cell wall components are necessary for the initiation of tuber development in the early stages. Li et al. (2019) found that pectinesterase was up-regulated in the initiation of *P. notoginseng* taproot thickening [36].

DEGs encoding galacturonosyltransferase (GAUTs) and peroxidase were up-regulated in the rapid swelling stage (C02). GAUTs are enzymes responsible for catalyzing glycosylation reactions and are closely related to the pectin and cellulose biosynthesis [50,51]. In plant development, GAUT1 is involved in pectin synthesis, and GAUT13 and GAUT14 could promote the synthesis of pectin and xylan in pollen tube walls and vegetative cell walls [52,53]. Peroxidase is an important antioxidant for scavenging reactive oxygen species in plants [54]. In addition, peroxidase has specific functions in cell wall formation and, in particular, lignin biosynthesis in *Selaginella martensii* [55]. Passardi et al. (2006) found that two peroxidases (AtPrx33 and Atprx34) promoted cell elongation, and this most likely occurs within the cell walls in *Arabidopsis thaliana* [56]. In the previous study, peroxidase was related to cell elongation and compound crosslinking of cell walls during the *Euphorbia kansui* laticifer developmental process [57]. The highest level of peroxidase and GAUT genes was found in the rapid swelling stage (C02). All these suggested that they might be related to the cell wall component metabolism during tuber swelling.

## 5. Conclusions

The tuber is the medicinal organ of *C. yanhusuo*. The full-length transcriptome and comparative transcriptome at three critical developmental stages of *C. yanhusuo* tuber were investigated in our study. A total of 90,496 full-length non-chimeric transcripts were obtained, and 9221 DEGs were identified during the tuber swelling process. Sucrose metabolism and starch accumulation are necessary for tuber enlargement of *C. yanhusuo*. Zeatin may play a role in the tuber formation stage, while brassinosteroids may play a role in the later stage of tuber swelling. Furthermore, peroxidase, pectinesterase, and galacturonosyltransferase were involved in cell wall metabolism and modification in the tuber swelling process. The swelling of the *C. yanhusuo* tuber is determined by many factors, including plant hormone signal transduction and metabolism processes. All these results provide the basis to clarify the molecular biological mechanism of tuber formation in *C. yanhusuo* and to further cultivate large tuber varieties.

## Figures and Tables

**Table 1:** The statistical analysis of *Corydalis yanhusuo* SMRT sequencing.

CCS Number	Read Bases of CCS	Mean Read Length of CCS	FLNC Reads Number	Consensus Isoforms Number	Average Consensus Isoforms Length	Polished High-Quality Isoforms Number
136,469	288,681,328	2115	90,496	36,347	1795	35,513

Note: CCS: circular consensus sequences; FLNC: full-length non-chimeric.

**Table 2:** Functional annotation of *Corydalis yanhusuo* unigenes.

Database	Number of Unigenes	300 ≤ Length < 1000	Length ≥ 1000
Nr	19,252	3055	16,103
Swiss-prot	14,427	2090	12,278
Pfam	15,860	2054	13,798
GO	13,506	2165	11,301
COG	8172	945	7219
KOG	12,663	1827	10,797
KEGG	9324	1520	7764
All	19,341	3095	16,143

**Table 3:** Summary of *Corydalis yanhusuo* tubers second-generation sequencing data.

Sample	Read Number	Base Number	GC Content (%)	Q30 Percentage (%)
C01-1	20,187,733	6,047,288,820	43.90	93.06
C01-2	22,295,014	6,674,832,134	43.90	93.20
C01-3	24,651,686	7,382,052,386	43.96	93.09
C02-1	19,850,543	5,946,722,130	43.43	92.87
C02-2	19,876,319	5,954,700,720	43.44	93.49
C02-3	20,915,770	6,264,762,952	43.43	93.28
C03-1	20,088,356	6,020,004,766	43.44	92.72
C03-2	21,393,807	6,411,557,256	43.38	93.24
C03-3	21,930,778	6,570,423,706	43.43	92.34

Note: C01: the tuber at initial expanding stage; C02: the tuber at rapid swelling stage; C03: the tuber at maturation stage.

**Table 4:** Function annotation of differential expression of unigenes in public database.

Sample Pairs	Annotated	COG	GO	KEGG	KOG	Pfam	Swiss-Prot	eggNOG	Nr
C01 vs. C02	3223	1511	2271	1321	1805	2748	2539	3134	3208
C01 vs. C03	4453	2122	3240	2168	2743	3733	3539	4322	4430
C02 vs. C03	7649	3487	5519	3634	821	6466	5938	7441	7620

Note: C01: the tuber at initial expanding stage; C02: the tuber at rapid expanding stage; C03: the tuber at maturation stage.

## Data Availability

The raw data were deposited in the NCBI Sequence Read Archive (SRA) with accession number PRJNA838804.

## Supplementary Materials

The following supporting information can be downloaded at https://www.mdpi.com/article/10.3390/life13112207/s1. Figure S1: The morphological structure of the tuber at the initial expanding stage (a) and rapid swelling stage (b). Figure S2: The sequencing quality of each sample with three replicates. Figure S3: The Venn diagram of common unigenes in three pairwise. Figure S4: The expression levels determined by qRT-PCR and RNA-seq from three stages. a: The expression levels of 8 genes by qRT-PCR; b: The expression levels of 8 genes by RNA-seq. 1: peroxidase, 2: beta-amylase, 3: galacturonosyltransferase, 4: pectinesterase, 5: auxin response factor, 6: zeatin O-glucosyltransferase, 7: 1,4-alpha-glucan-branching enzyme, 8: alpha-1,4 glucan phosphorylase. Figure S5: Gene ontology functional classification of differentially expressed genes. Figure S6: Expression patterns of unigenes related to starch and sucrose metabolism. C01: initial expanding stage; C02: rapid swelling stage; C03: maturation stage. Figure S7: Expression patterns of unigenes related to plant hormone signaling transduction. C01: initial expanding stage; C02: rapid swelling stage; C03: maturation stage. Table S1: The common differentially expressed genes of three developmental stages in *Corydalis yanhusuo* tuber. Table S2: Primers for qRT-PCR validation of selected genes.
