# Supplementary material for: Integrating Full-Length and Second-Generation Transcriptomics to Reveal Differentially Expressed Genes Associated with the Development of Corydalis yanhusuo Tuber
Source: Life (Basel). 2023 Nov 14;13(11):2207. doi: 10.3390/life13112207 (PMC10672666; doi:10.3390/life13112207)
Supplement: Supplementary file 1 [file life-13-02207-s001.zip › Supplementary tables/Table S2.pdf]

Table S2 Primers for qRT-PCR validation of selected genes.

Gene code	Gene ID	Primer sequence
actin	AT1G49240	F: GGTAACATTGTGCTCAGTGGTGG
		R: AACGACCTTAATCTTCATGCTGC
1	transcript_14664	F: GCCCTGAAGTTGTTTCTTGTGCT
		R: TGCACGGCTTCTCCTTCCAT
2	transcript_33342	F: ACGTGAACCATGCTGCGGA
		R: CCTCTGCCCTTTGTTCCGAGT
3	transcript_24801	F: GCCATGGTCACCCTCTTCGA
		R: TGCTTGGGTATGCTGCTCGA
4	transcript_10739	F: ATGAGAGCTCCTCCCATGGC
		R: TGTTGAGAGCCCATGTTGTTGA
5	transcript_10085	F: AGCGGCACCTTCTCACAAGT
		R: CACGTCTCACTCCCAACCGT
6	transcript_18936	F: TCCCGTCTCATTTCCACCCG
		R: CACGTTCCTTTGCTTGACGGT
7	transcript_593	F: GGCGACAAGACCATTGCGTT
		R: CCCTCACCTCCCAATGCCAT
8	transcript_10565	F: GGGAGCATGTTAAAGGAGCCG
		R: AGAGAAGCAGAGCACAGCGT
