# Supplementary figures and images for: Integrating Full-Length and Second-Generation Transcriptomics to Reveal Differentially Expressed Genes Associated with the Development of Corydalis yanhusuo Tuber
Source: Life (Basel). 2023 Nov 14;13(11):2207. doi: 10.3390/life13112207 (PMC10672666; doi:10.3390/life13112207)

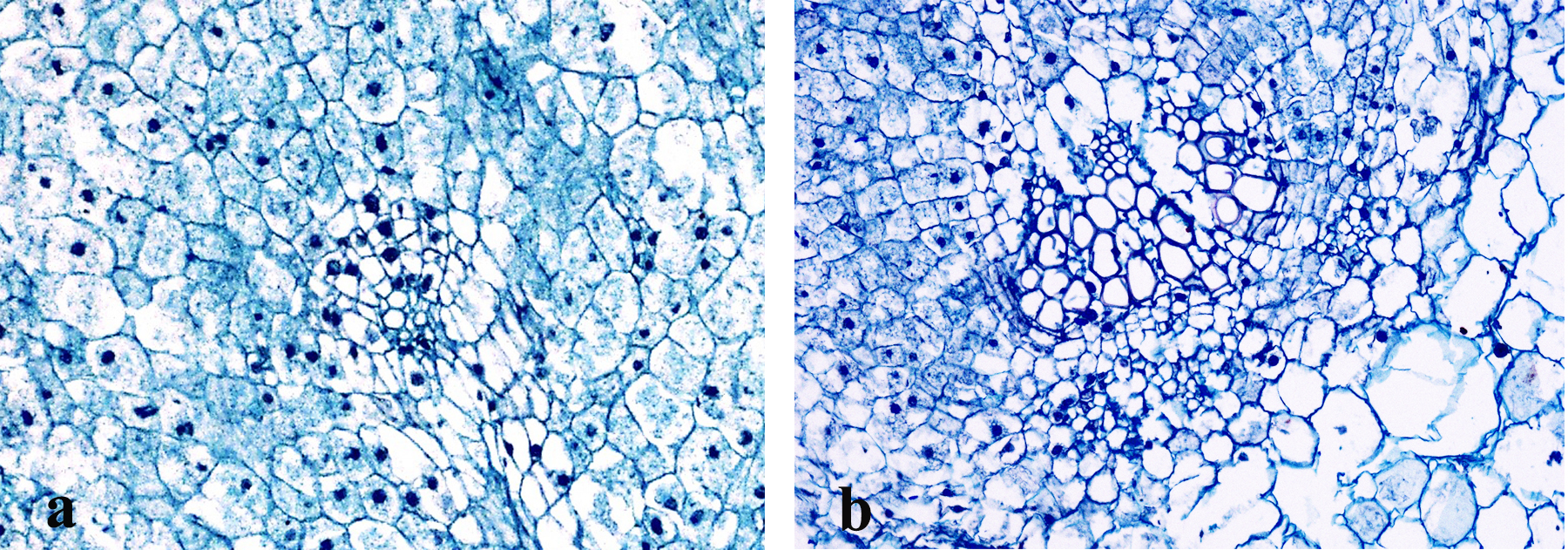

Supplement: Supplementary file 1 [file life-13-02207-s001.zip › Supplementary Figures/Figure S1.jpg]

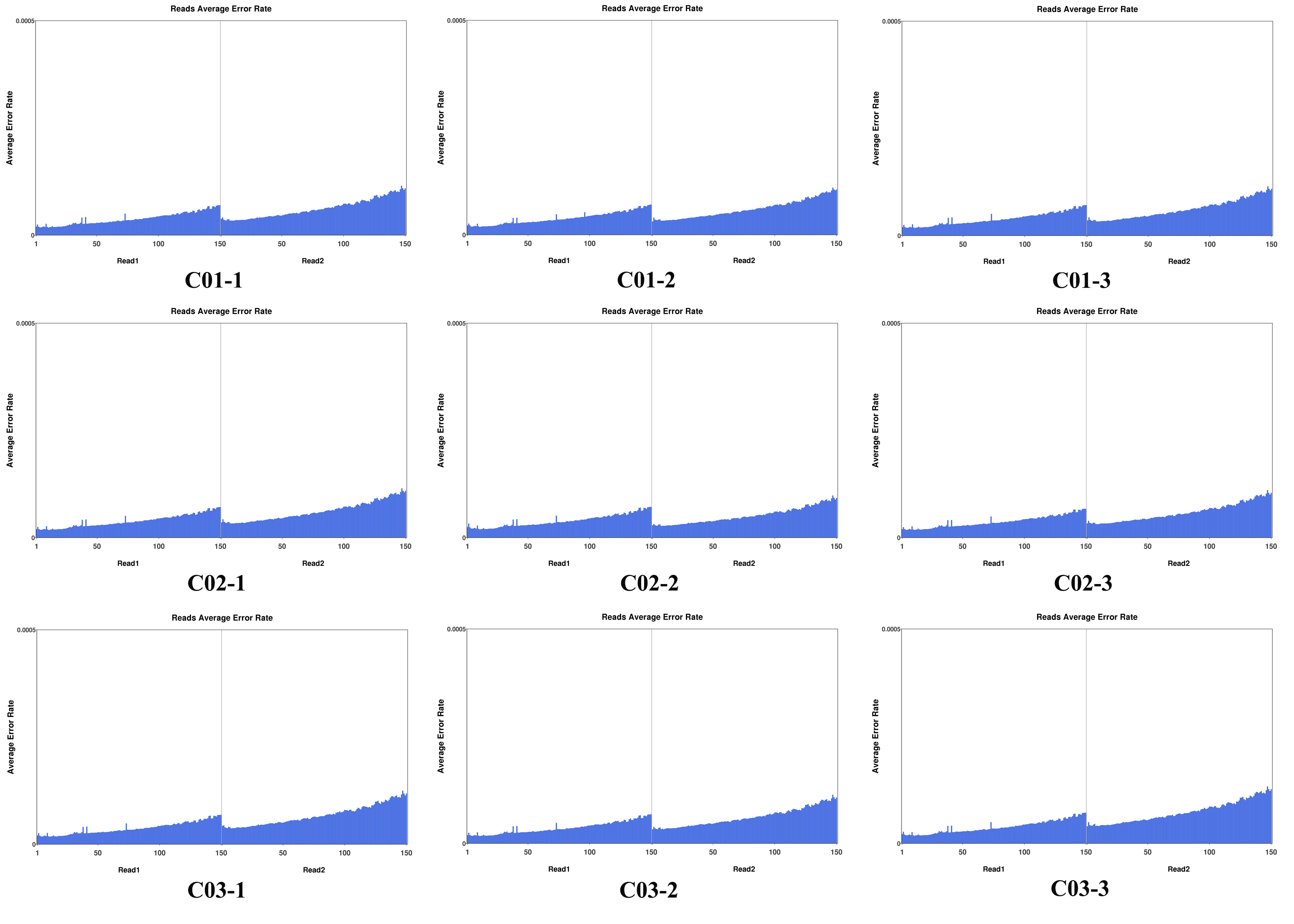

Supplement: Supplementary file 1 [file life-13-02207-s001.zip › Supplementary Figures/Figure S2.jpg]

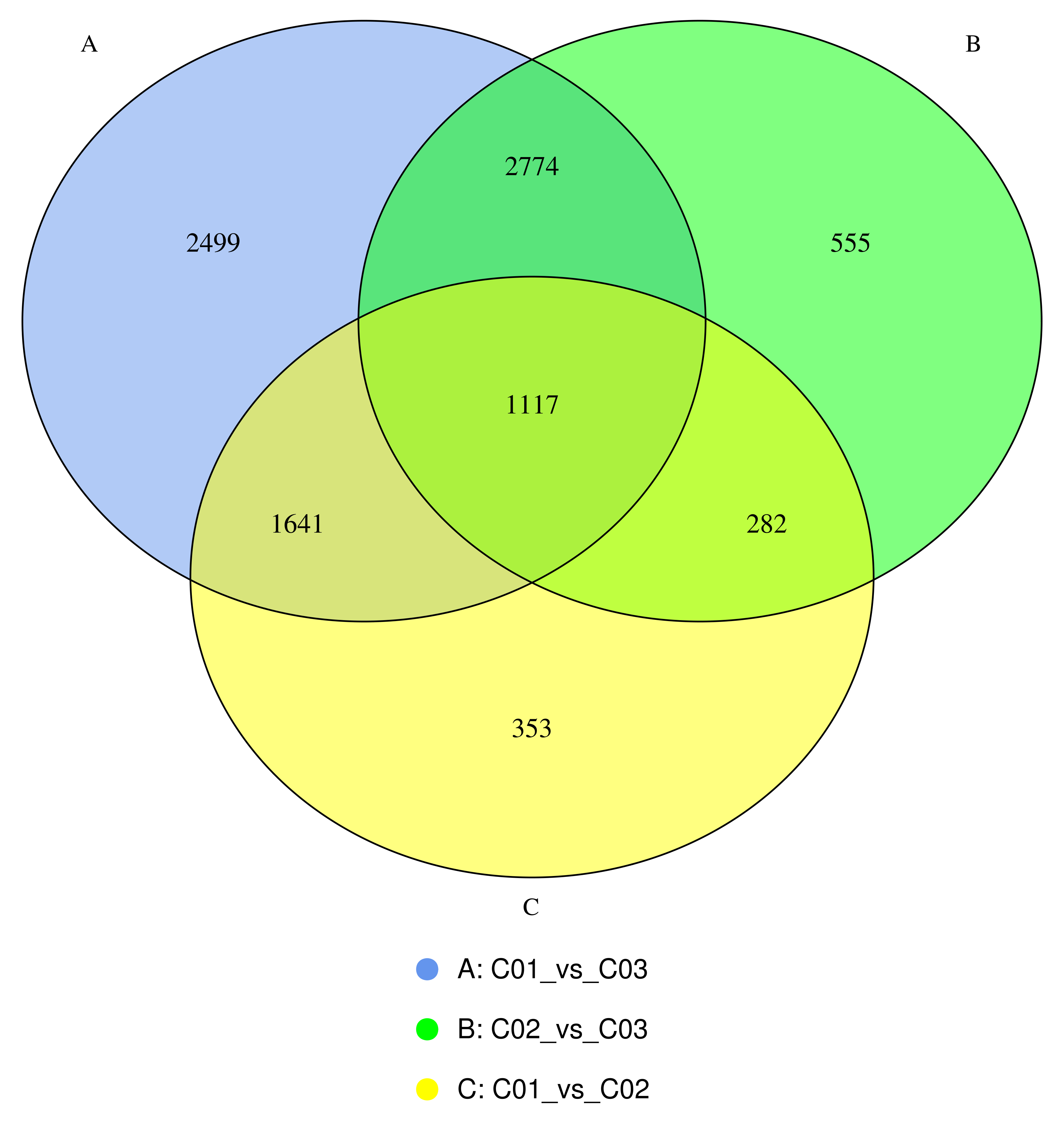

Supplement: Supplementary file 1 [file life-13-02207-s001.zip › Supplementary Figures/Figure S3.png]

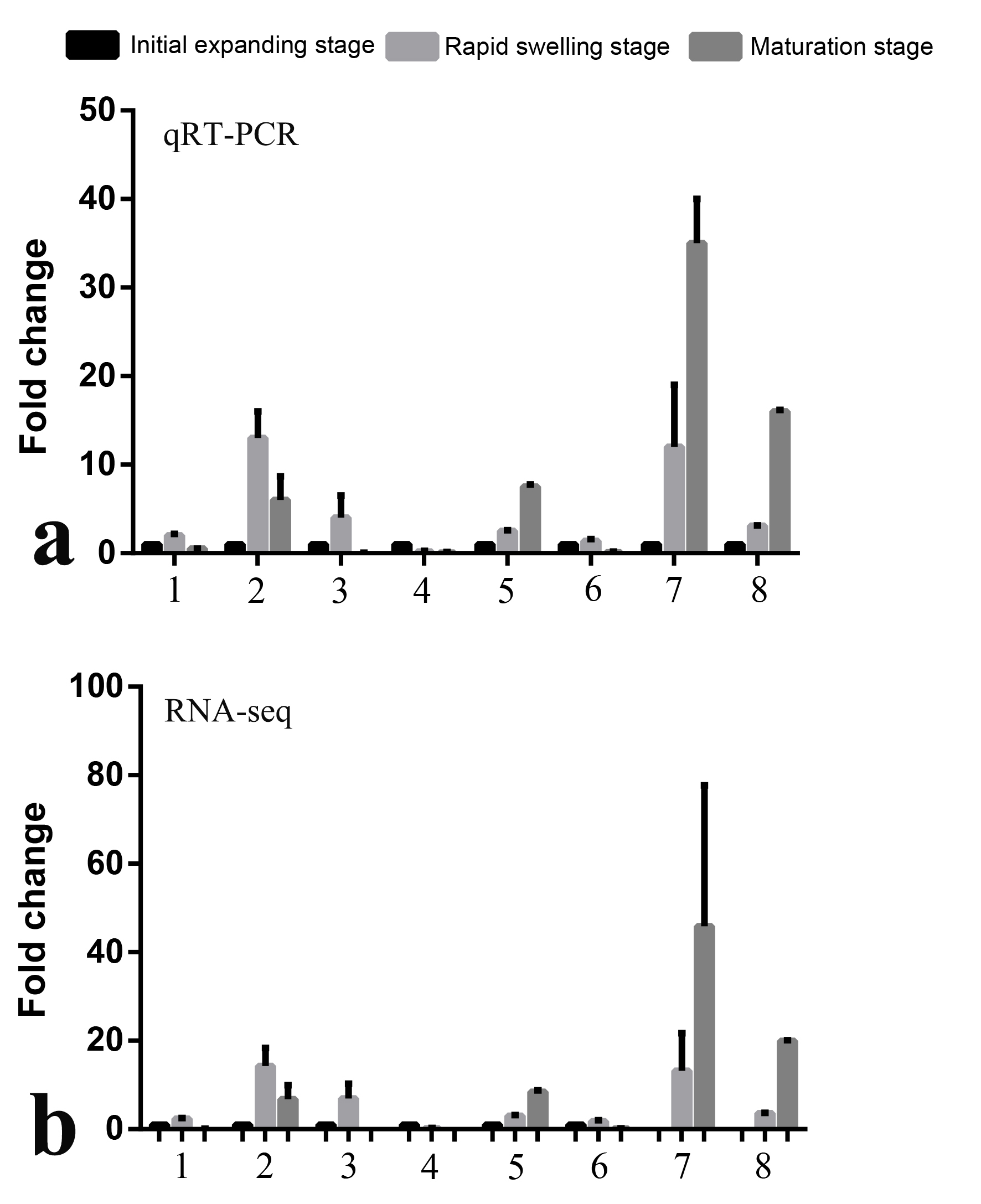

Supplement: Supplementary file 1 [file life-13-02207-s001.zip › Supplementary Figures/Figure S4.jpg]

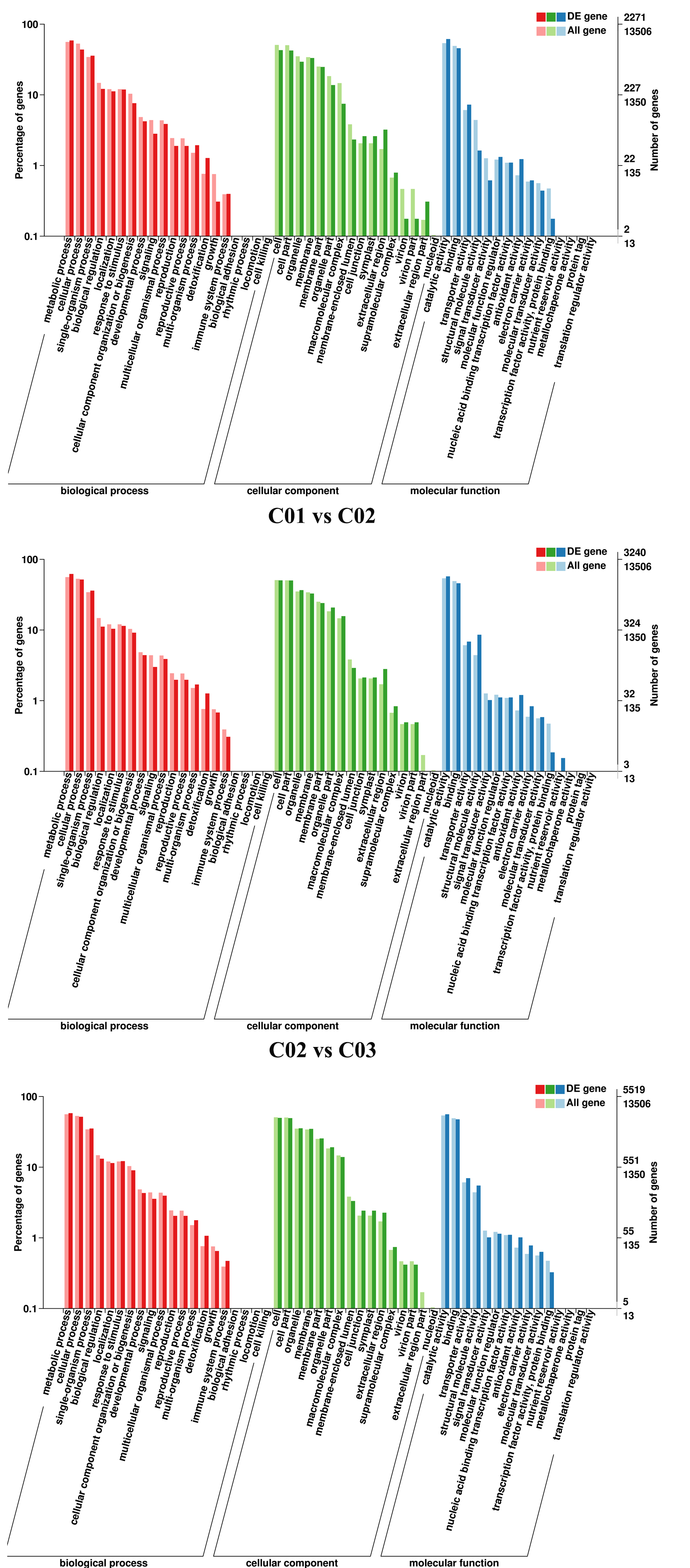

Supplement: Supplementary file 1 [file life-13-02207-s001.zip › Supplementary Figures/Figure S5.jpg]

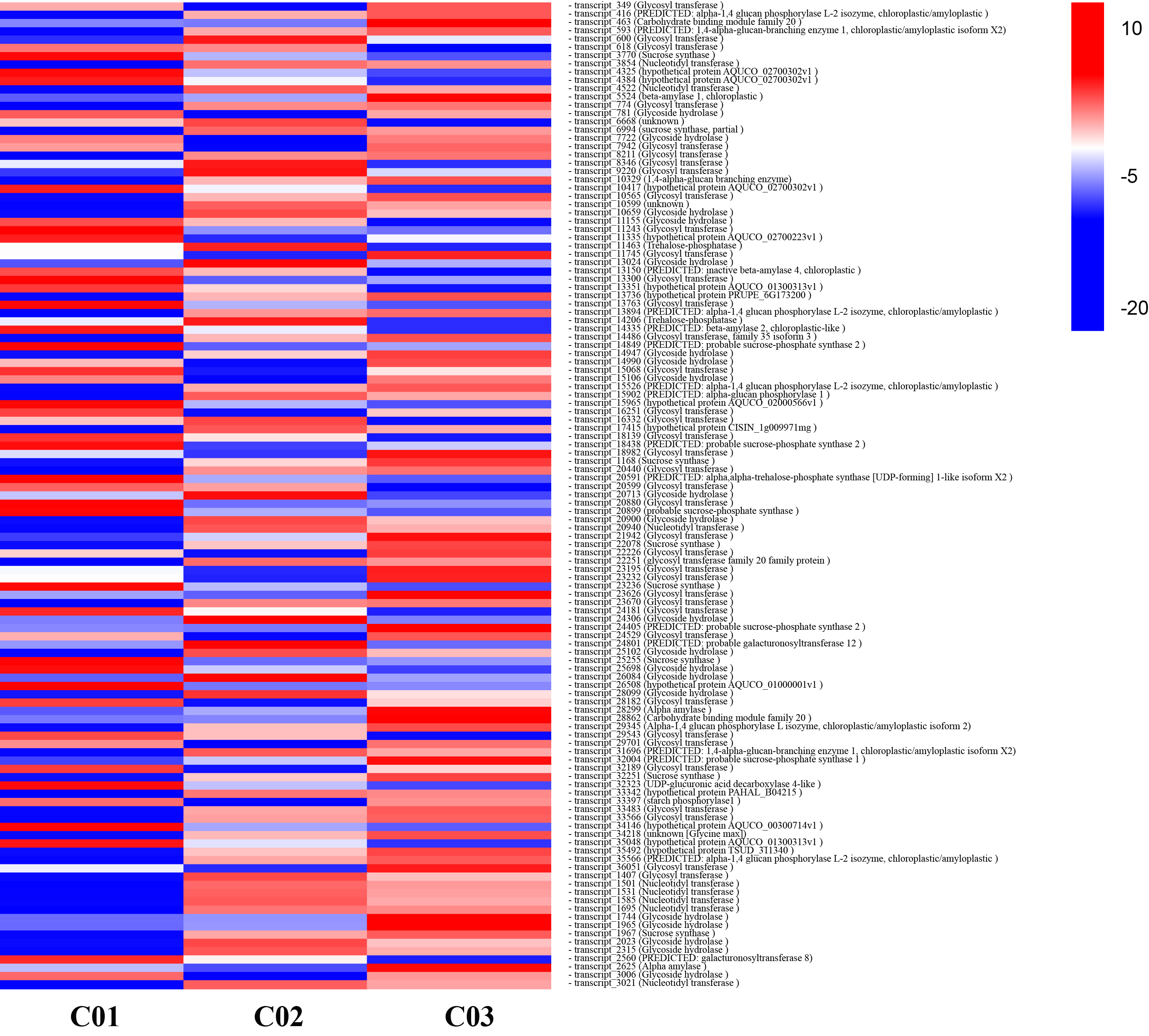

Supplement: Supplementary file 1 [file life-13-02207-s001.zip › Supplementary Figures/Figure S6.tif]

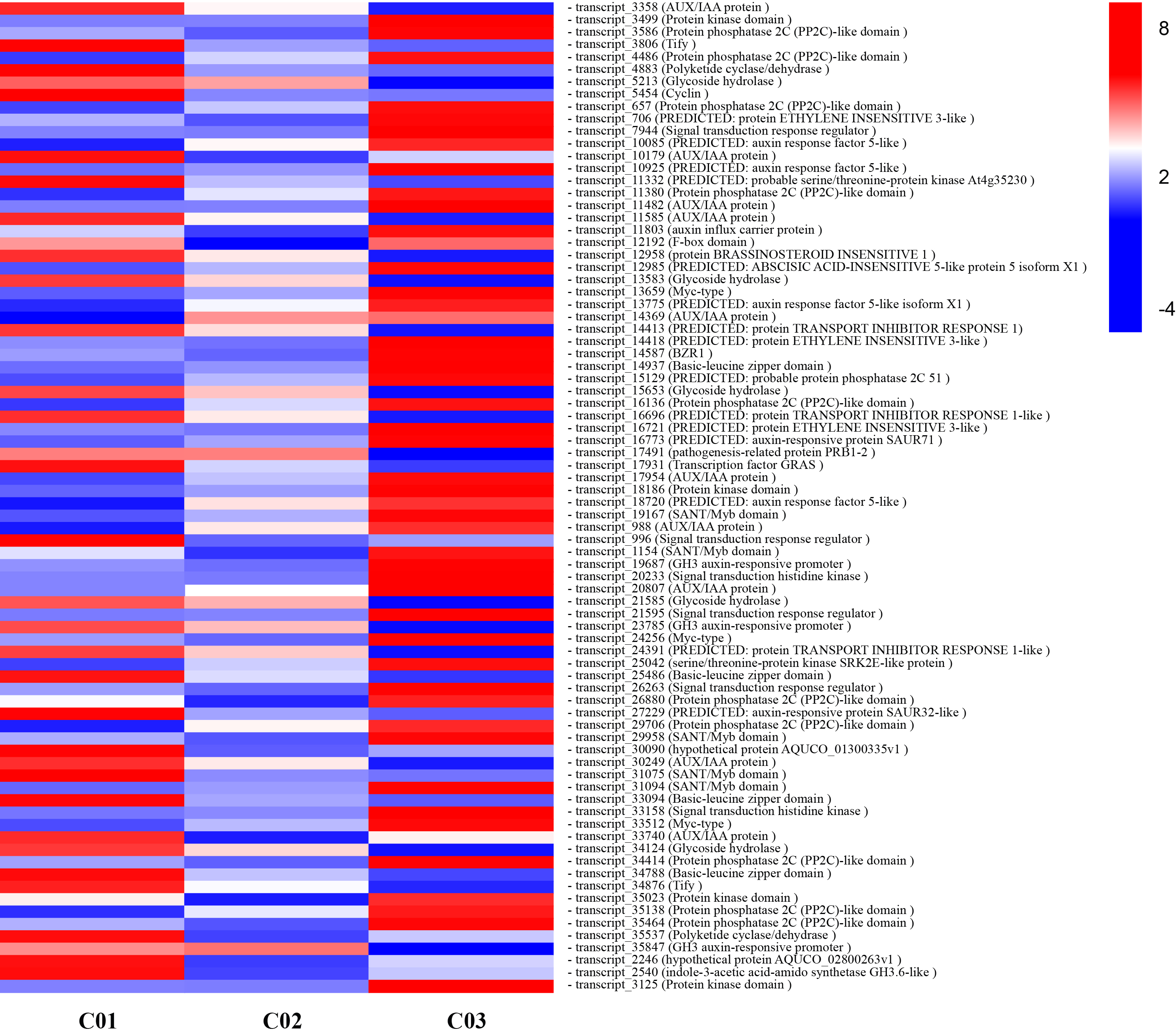

Supplement: Supplementary file 1 [file life-13-02207-s001.zip › Supplementary Figures/Figure S7.tif]
